# Prognostic Value of Pretreatment Prognostic Nutritional Index in Patients With Renal Cell Carcinoma: A Meta-Analysis

**DOI:** 10.3389/fonc.2021.719941

**Published:** 2021-10-05

**Authors:** Changqing Mao, Weixin Xu, Weina Ma, Chun Wang, Zhaojiao Guo, Jun Yan

**Affiliations:** ^1^ Department of Nephrology, Jiading District Central Hospital Affiliated Shanghai University of Medicine & Health Sciences, Shanghai, China; ^2^ Department of Laboratory Medicine, Jiading District Central Hospital Affiliated Shanghai University of Medicine & Health Sciences, Shanghai, China; ^3^ Department of Pharmacy, Jiading District Central Hospital Affiliated Shanghai University of Medicine & Health Sciences, Shanghai, China; ^4^ Department of Oncology, Jiading District Central Hospital Affiliated Shanghai University of Medicine & Health Sciences, Shanghai, China

**Keywords:** PNI, renal cell carcinoma, meta-analysis, prognosis, immune responses

## Abstract

**Background:**

The pretreatment prognostic nutritional index (PNI) is correlated with poor prognosis in several malignancies. However, the prognostic role of PNI in patients with renal cell carcinoma (RCC) remains unclear. Therefore, we performed a meta-analysis to investigate the prognostic significance of PNI in patients with RCC.

**Methods:**

We searched the PubMed, Web of Science, Embase, Scopus, and Cochrane Library databases up to February 2021. Pooled hazard ratios (HRs) and 95% confidence intervals (CIs) were used to estimate correlation between PNI and survival endpoints in RCC.

**Results:**

Ten studies with 4,908 patients were included in the meta-analysis. The pooled results indicated that a low PNI associated with poor overall survival (HR = 2.10, 95% CI = 1.67–2.64, p<0.001), shorter progression-free survival, disease-free survival, recurrence-free survival (HR = 1.99, 95% CI = 1.67–2.36, p<0.001), and poor cancer-specific survival (HR = 2.95, 95% CI = 1.61–5.39, p<0.001). Additionally, the prognostic ability of PNI was not affected by subgroup analysis factors.

**Conclusion:**

The meta-analysis indicated that low PNI associated with shorter survival outcomes in patients with RCC. Therefore, PNI could be used as an effective prognostic indicator in RCC.

## Introduction

Renal cell carcinoma (RCC) is the most prevalent form of kidney tumor, accounting for 85% of cases (1). RCC is the most lethal urological malignancy and is responsible for approximately 2%–3% of all adult malignancies (2). Surgical resection, including partial and radical nephrectomy, is a treatment with curative intent in patients with localized RCC (3). A majority of the patients with RCC are diagnosed at the localized stage, but 1/3 patients present with locally advanced or metastatic status (4). Moreover, >25% of patients with localized disease show metastatic progression after the initial treatment. Furthermore, the prognosis of patients with advanced disease is dismal, with a 5-year survival rate of 11% (5). Thus, prognostic scores and parameters would be helpful in determining the survival of patients with RCC (6).

Patients with cancer usually experience malnutrition and changes in immune responses during disease development (7). The prognostic nutritional index (PNI) is evaluated according to serum albumin levels and lymphocyte count in the peripheral blood (8). PNI reflects the nutritional and immunologic status of patients with cancer and is a prognostic factor in several solid tumors (9). Low PNI is associated with poor prognosis in some cancers, such as pancreatic (10), lung (11), esophageal cancer (EC) (12), and ovarian (13) cancers, and nasopharyngeal carcinoma (14). Many studies have also explored the prognostic significance of PNI in patients with RCC; however, the results have been inconsistent (15–24). For example, some studies identified low PNI as a significant prognostic factor for RCC (20, 22), whereas others failed to detect the prognostic role of PNI in RCC (17). Therefore, in this study, we performed a meta-analysis to quantitatively evaluate association between PNI and prognosis in patients with RCC.

## Materials and Methods

### Literature Retrieval

The meta-analysis was performed under the guidance of the Preferred Reporting Items for Systematic Reviews and Meta-Analyses (PRISMA) statement (25). We searched PubMed, Web of Science, Embase, Scopus, and Cochrane Library databases up to February 2021. The search was performed using following terms: “prognostic nutritional index,” “PNI,” “kidney cancer,” “renal cell carcinoma,” “renal tumor,” and “renal neoplasms.” Only studies published in English were considered. Additionally, literature references were manually screened to identify potentially relevant studies. No ethical approval or informed consent was required as all data were based on previously published articles.

### Selection Criteria

Studies fulfilling following features were eligible for the meta-analysis: 1) studies using pathological methods to confirm RCC; 2) studies reporting hazard ratios (HRs) and corresponding 95% confidence intervals (CIs) for estimating associations between PNI and survival outcomes or had sufficient data to calculate these statistics; 3) identified a cutoff value to stratify low and high PNI; and 4) were full-text articles. The exclusion criteria were as follows: 1) reviews, case reports, meeting abstracts, letters, and comments; 2) studies with no data of interest for this meta-analysis; 3) animal studies; and 4) non-English studies.

### Data Extraction

The data were extracted by two independent investigators (C.M. and W.X.), and all disagreements were resolved by discussion with a third researcher (J.Y.). The following information was extracted: name of the first author, country of origin, ethnicity, sample size, age, study duration, metastatic status of disease, Fuhrman grade, histological type, treatment methods, cutoff value of PNI, cutoff value determination methods, survival endpoints, survival analysis types, and HRs with 95% CIs. The primary study outcome was overall survival (OS), and secondary study outcomes were progression-free survival (PFS), disease-free survival (DFS), recurrence-free survival (RFS), and cancer-specific survival (CSS). PFS/DFS/RFS were combined because survival was calculated based on the duration of event-free survival. Moreover, this combination of PFS/DFS/RFS was based on previous studies on PNI (26, 27).

### Quality Assessment

Two researchers (W.M. and C.W.) independently evaluated the methodological quality of eligible studies according to the Newcastle-Ottawa Scale (NOS) (28). The NOS was used to assess the quality based on three aspects: selection of subjects (four stars), comparability of study groups (two stars), and outcome measurement (three stars). The NOS ranged from 0 to 9, and studies with NOS ≥6 were considered high-quality.

### Statistical Analysis

Pooled HRs and 95% CIs were used to estimate correlations between PNI and survival endpoints. The heterogeneity among studies was evaluated using Cochran’s Q and *I* ^2^ statistics. *I* ^2^>50% and P for heterogeneity <0.10 indicated significant heterogeneity; and a random-effects model (REM) was applied. However, a fixed-effects model (FEM) was adopted otherwise. Subgroup analysis was performed to further investigate the prognostic role of PNI in various patient groups. Sensitivity analysis was performed to explore the impact of each study on the overall pooled results of the meta-analysis. Publication bias was estimated by visual inspection of the Begg’s funnel plot. All statistical analyses were performed using Stata software version 15.0 (STATA Corporation, College Station, TX, USA). A p < 0.05 was considered to be statistically significant.

## Results

### Search Results and Study Characteristics

**Figure 1:**
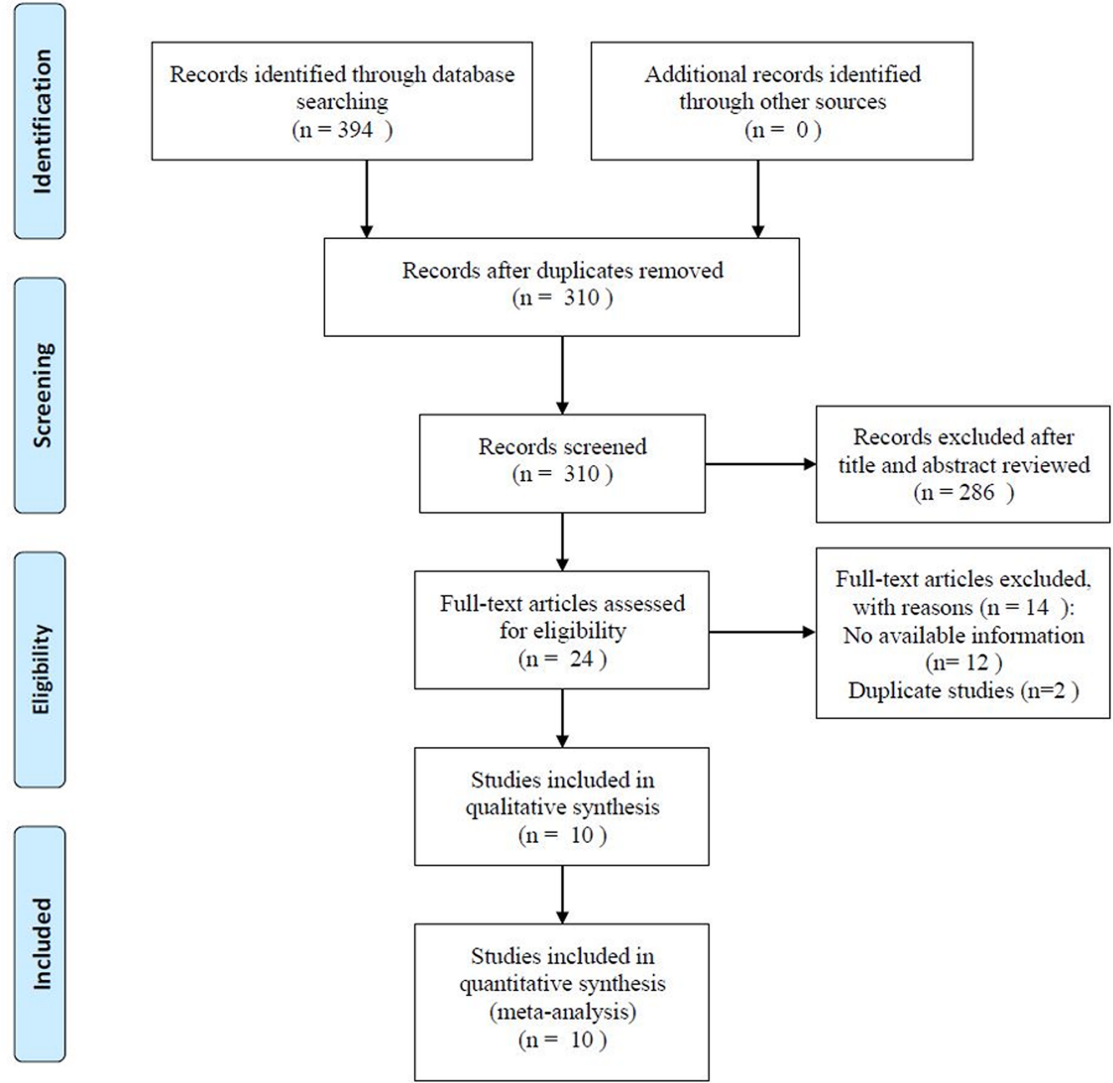
Flow chart of literature selection.

The process of literature selection and screening is shown in **Figure 1**. The initial literature search identified 394 records. After removing 84 duplicate records, 310 articles were screened. After reviewing the titles, abstracts, and full texts, 10 studies (15–24) were included in the meta-analysis. The basic characteristics of the included studies are listed in **Table 1**. The studies were published between 2015 and 2020, and were conducted in five countries, including China (n = 4) (16, 17, 22, 24), Korea (n = 3) (18–20), the USA (n = 1) (15), Austria (n = 1) (21), and Turkey (n = 1) (23). The total sample size was 4,908, ranging from 125 to 1,360 and with a median value of 413. Five studies enrolled patients with non-metastatic disease (15, 18, 19, 21, 24), three studies recruited patients with metastatic disease (16, 20, 23), and two studies enrolled patients with mixed disease stages (17, 22). All studies had a retrospective study design. Nine studies recruited patients with clear cell renal cell carcinoma (ccRCC) and non-clear cell renal cell carcinoma (nccRCC) (15–20, 22–24), and one study included patients with ccRCC (21). Regarding treatment methods, six studies applied partial or radical nephrectomy (15, 17, 19, 21, 22, 24), three studies administered tyrosine kinase inhibitors (TKIs) (16, 20, 23), and one study applied radical nephrectomy (18). The cutoff values for PNI ranged from 38.5 to 51.62, with a median value of 46.31. The NOS of all studies was >6, indicating that all eligible studies were of high quality. The detailed items for the NOS scores are shown in **Table 2**.

**Table 1:** Main characteristics of eligible studies in the meta-analysis.

Author	Year	Country	Ethnicity	No. of patients	Age (year)	Study duration	Metastatic status	Fuhrman grade	Histologytype	Treatment	Cut-off value	Cut-off determination	Survival endpoints	Survival analysis	Study design	NOS score
Broggi	2016	USA	Non-Asian	341	Median:61.5	2001-2014	Non-metastatic	G1-G2:151	ccRCC:341	Partial or radical nephrectomy	44.7	ROC analysis	OS, RFS	MVA	R	7
								G3-G4:190								
Cai	2017	China	Asian	178	Median:60	2006-2015	Metastatic	G1-G2:103	ccRCC:170	TKIs	51.62	ROC analysis	OS, PFS	MVA	R	8
					Range:24-82			G3-G4:60	nccRCC:8							
								Unknown:15								
Hu	2020	China	Asian	660	Mean:54.89	2010-2013	Mixed	G1-G2:356	ccRCC:558	Partial or radical nephrectomy	44.3	X-tile program	OS, PFS, CSS	MVA	R	7
								G3-G4:304	nccRCC:102							
Kang	2017	Korea	Asian	324	Median:55	1996-2012	Non-metastatic	G1-G2:84	ccRCC:278	Radical nephrectomy	45	ROC analysis	OS, CSS	MVA	R	8
					Range:48-64			G3-G4:237	nccRCC:43							
Kim	2020	Korea	Asian	459	Mean:55.8	1994-2017	Non-metastatic	G1-G2:176	ccRCC:398	Partial or radical nephrectomy	51	ROC analysis	RFS, CSS	MVA	R	8
					Range:18-81			G3-G4:283	nccRCC:61							
Kwon	2017	Korea	Asian	125	Median:58	2007-2014	Metastatic	G1-G2:30	ccRCC:102	TKIs	41	Cox model	OS, PFS	UVA	R	7
								G3-G4:55	nccRCC:15							
								Unknown:40	Unknown:8							
Lucca	2015	Austria	Non-Asian	430	Median:65.5	2002-2014	Non-metastatic	G1-G2:346	ccRCC:430	Partial or radical nephrectomy	48	Cox model	DFS	MVA	R	9
								G3-G4:84								
Peng	2017	China	Asian	1360	Mean:55	2001-2010	Mixed	G1-G2:1103	ccRCC:1228	Partial or radical nephrectomy	47.62	ROC analysis	OS, PFS	MVA	R	8
					Range:14-87			G3-G4:257	nccRCC:132							
Yasar	2020	Turkey	Asian	396	Mean:58	2007-2017	Metastatic	NR	ccRCC:295	TKIs	38.5	Median value	OS	UVA	R	7
					Range:29-88				nccRCC:63							
Zheng	2018	China	Asian	635	Mean:61.7	2004-2014	Non-metastatic	G1-G2:472	ccRCC:559	Partial or radical nephrectomy	48	Cox model	OS, CSS	UVA	R	7
								G3-G4:163	nccRCC:76							

ccRCC, clear cell renal cell carcinoma; nccRCC, non- clear cell renal cell carcinoma; OS, overall survival; RFS, recurrence-free survival; PFS, progression-free survival; DFS, disease-free survival; CSS, cancer-specific survival; TKIs, tyrosine kinase inhibitors; NR, not reported; ROC, receiver operating characteristic; MVA, multivariate; UVA, univariate; NOS, Newcastle-Ottawa Scale; R, retrospective.

**Table 2:** Quality assessment conducted according to the NOS for all included studies.

Author	Year	Selection				Comparability	Outcome			NOS score
		Representativeness of the exposed cohort	Selection of the non-exposed cohort	Ascertainment of exposure	Demonstration that outcome of interest was not present at start of study	Comparability of cohorts on the basis of the design or analysis	Assessment of outcome	Was follow-up long enough for outcomes to occur	Adequacy of follow up of cohorts	
Broggi	2016	★	★	★	★	★	★	–	★	7
Cai	2017	–	★	★	★	★★	★	★	★	8
Hu	2020	★	★	★	★	★	★	★	–	7
Kang	2017	★	★	★	★	★★	★	–	★	8
Kim	2020	★	★	★	★	★★	★	★	–	8
Kwon	2017	★	–	★	★	★	★	★	★	7
Lucca	2015	★	★	★	★	★★	★	★	★	9
Peng	2017	★	★	★	★	★★	★	★	–	8
Yasar	2020	★	★	★	★	★	★	–	★	7
Zheng	2018	–	★	★	★	★	★	★	★	7

NOS, Newcastle-Ottawa Scale. A star represents one point.

### PNI and OS in RCC

**Figure 2:**
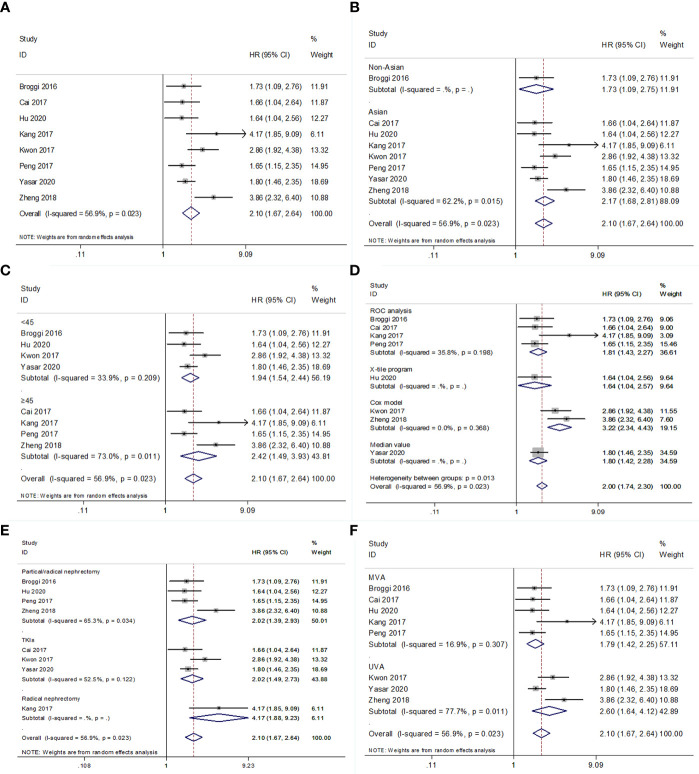
Forest plot examining the association between PNI and OS in patients with RCC. **(A)** overall patient population; **(B)** subgroup analysis by various ethnicities; **(C)** subgroup analysis by various cut-off values of PNI; **(D)** subgroup analysis by various cut-off value determination methods; **(E)** subgroup analysis by various treatment methods; **(F)** subgroup analysis by various survival analysis types.

Eight studies (15–18, 20, 22–24) with 4,019 patients investigated the association between low PNI and OS in patients with RCC. The heterogeneity was significant (*I* ^2^ = 56.9%, P=0.023); therefore, REM was applied. The pooled results had HR = 2.10, 95% CI = 1.67–2.64, p<0.001 (**Figure 2** and **Table 3**). Subgroup analyses were performed according to ethnicity, cutoff value, cutoff value determination method, treatment, and survival analysis type. The REM and FEM were selected according to the heterogeneity in each subgroup. As shown in **Figure 2**; **Supplementary Figure 1** and **Table 3**, a low PNI was a significant prognostic factor in all subgroups (p<0.05). The results indicated that reduced PNI correlated with poor OS, and that the prognostic role was not influenced by subgroup factors.

**Table 3:** Results of subgroup meta-analysis for overall survival.

Variables	No. of studies	No. of patients	Effects model	HR (95%CI)	p	*I* ^2^ (%)	P for heterogeneity
Total	8	4,019	REM	2.10 (1.67-2.64)	<0.001	56.9	0.023
Ethnicity							
Asian	7	3,678	REM	2.17 (1.68-2.81)	<0.001	62.2	0.015
Non-Asian	1	341	–	1.73 (1.09-2.75)	0.021	–	–
Cut-off value							
<45	4	1,522	FEM	1.92 (1.61-2.28)	<0.001	33.9	0.209
≥45	4	2,497	REM	2.42 (1.49-3.93)	<0.001	73.0	0.011
Cut-off value determination							
ROC analysis	4	2,203	FEM	1.81 (1.43-2.27)	<0.001	35.8	0.198
Cox model	2	760	FEM	3.22 (2.34-4.43)	<0.001	0	0.368
X-tile program	1	660	–	1.64 (1.04-2.57)	0.031	–	–
Median value	1	396	–	1.80 (1.42-2.28)	<0.001	–	–
Treatment							
Partial or radical nephrectomy	4	2,996	REM	2.02 (1.39-2.93)	<0.001	65.3	0.034
Radical nephrectomy	1	324	–	4.17 (1.88-9.23)	<0.001	–	–
TKIs	3	699	REM	2.02 (1.49-2.73)	<0.001	52.5	0.122
Survival analysis							
MVA	5	2,863	FEM	1.77 (1.44-2.17)	<0.001	16.9	0.307
UVA	3	1,156	REM	2.60 (1.64-4.12)	<0.001	77.7	0.011
Metastatic status							
Non-metastatic	3	1,300	REM	2.91 (1.60-5.29)	<0.001	69.5	0.038
Metastatic	3	699	REM	2.02 (1.49-2.73)	<0.001	52.5	0.122
Mixed	2	2,020	FEM	1.64 (1.24-2.71)	<0.001	0	0.990
Histology							
ccRCC+nccRCC	7	2,678	REM	2.17 (1.68-2.81)	<0.001	62.2	0.015
ccRCC	1	341	–	1.73 (1.09-2.75)	0.021	–	–

TKIs, tyrosine kinase inhibitors; ROC, receiver operating characteristic; MVA, multivariate; UVA, univariate; FEM, fixed-effects model; REM, random-effects model; ccRCC, clear cell renal cell carcinoma; nccRCC, non- clear cell renal cell carcinoma.

### PNI and PFS/DFS/RFS in RCC

**Figure 3:**
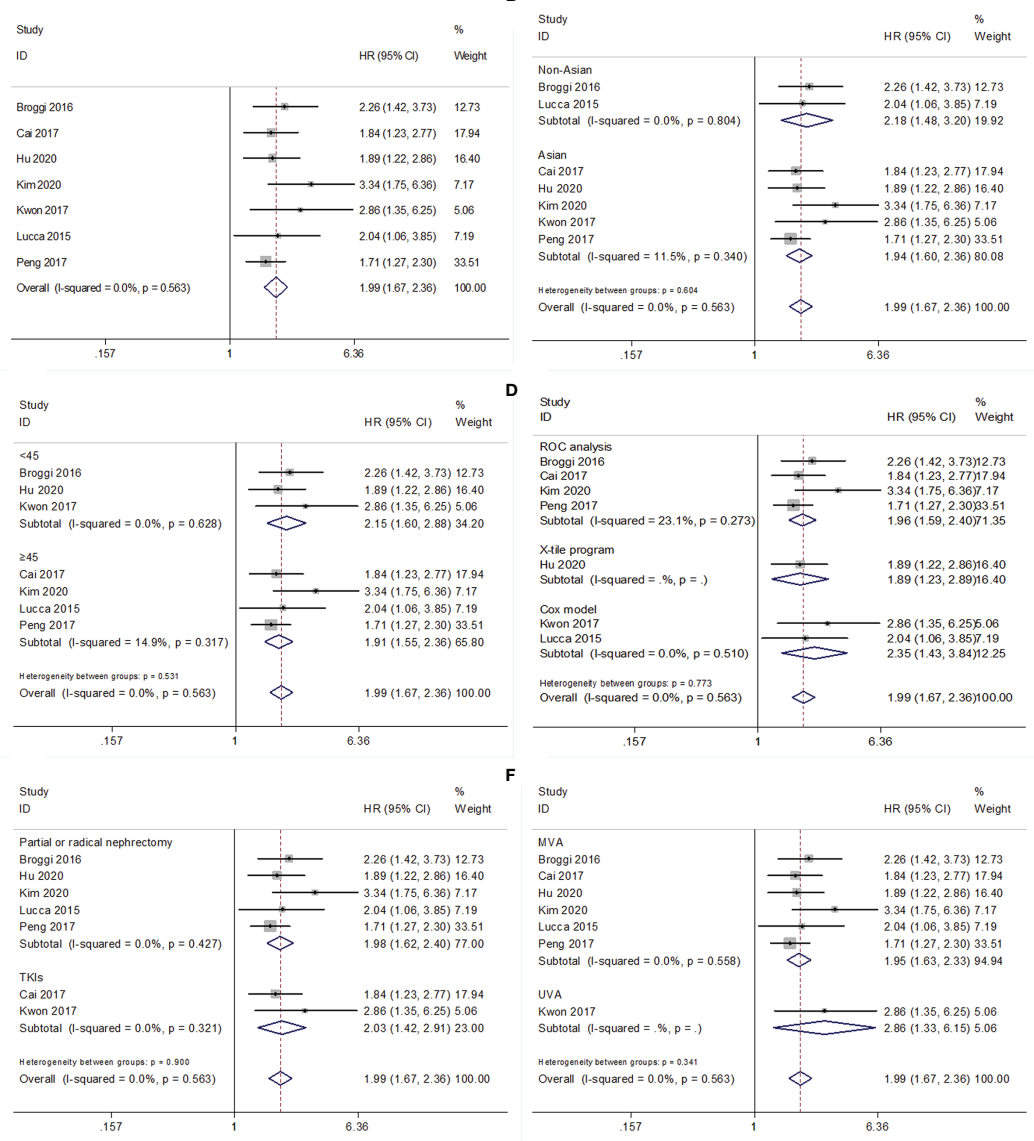
Forest plot examining the association between PNI and PFS/DFS/RFS in patients with RCC. **(A)** overall patient population; **(B)** subgroup analysis by various ethnicities; **(C)** subgroup analysis by various cut-off values of PNI; **(D)** subgroup analysis by various cut-off value determination methods; **(E)** subgroup analysis by various treatment methods; **(F)** subgroup analysis by various survival analysis.

For PFS/DFS/RFS, the data from seven studies with 3,553 patients were combined (15–17, 19–22). The combined data had HR = 1.99, 95% CI = 1.67–2.36, p<0.001; and an FEM was applied due to non-significant heterogeneity (*I* ^2^ = 0, P=0.563) (**Figure 3** and **Table 4**). Subgroup analyses were also performed, and the results indicated that low PNI associated with worse PFS/DFS/RFS irrespective of ethnicity, cutoff value, cutoff determination method, treatment, metastatic status, histology, and survival analysis (**Figure 3**; **Supplementary Figure 2** and **Table 4**).

**Table 4:** Results of subgroup meta-analysis for progression-free survival/disease-free survival/recurrence-free survival.

Variables	No. of studies	No. of patients	Effects model	HR (95%CI)	p	*I* ^2^ (%)	P for heterogeneity
Total	7	3,553	FEM	1.99 (1.67-2.36)	<0.001	0	0.563
Ethnicity							
Asian	5	2,782	FEM	1.94 (1.60-2.36)	<0.001	11.5	0.340
Non-Asian	2	771	FEM	2.18 (1.48-3.20)	<0.001	0	0.804
Cut-off value							
<45	3	1,126	FEM	2.15 (1.60-2.88)	<0.001	0	0.628
≥45	4	2,427	FEM	1.91 (1.55-2.36)	<0.001	14.9	0.317
Cut-off value determination							
ROC analysis	4	2,338	FEM	1.96 (1.59-2.40)	<0.001	23.1	0.273
Cox model	2	555	FEM	2.35 (1.43-3.84)	0.001	0	0.510
X-tile program	1	660	–	1.89 (1.23-2.89)	0.003	–	–
Treatment							
Partial or radical nephrectomy	5	3,250	FEM	1.98 (1.62-2.40)	<0.001	0	0.427
TKIs	2	303	FEM	2.03 (1.42-2.91)	<0.001	0	0.321
Survival analysis							
MVA	6	3,428	FEM	1.95 (1.63-2.33)	<0.001	0	0.558
UVA	1	125	–	2.86 (1.33-6.15)	0.007	–	–
Metastatic status							
Non-metastatic	3	1,230	FEM	2.44 (1.75-3.40)	<0.001	0	0.520
Metastatic	2	303	FEM	2.03 (1.42-2.91)	<0.001	0	0.321
Mixed	2	2,020	FEM	1.76 (1.38-2.25)	<0.001	0	0.702
Histology							
ccRCC+nccRCC	5	2,782	FEM	1.94 (1.60-2.36)	<0.001	11.5	0.340
ccRCC	2	771	FEM	2.18 (1.48-3.20)	<0.001	0	0.804

TKIs, tyrosine kinase inhibitors; ROC, receiver operating characteristic; MVA, multivariate; UVA, univariate; FEM, fixed-effects model; REM, random-effects model; ccRCC, clear cell renal cell carcinoma; nccRCC, non- clear cell renal cell carcinoma.

### PNI and CSS in RCC

**Figure 4:**
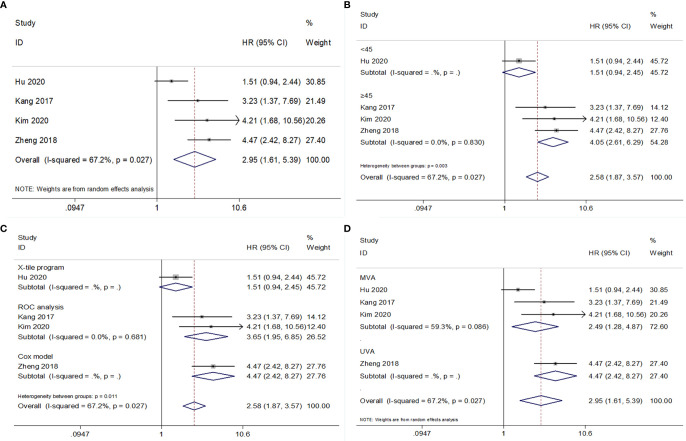
Forest plot examining the association between PNI and CSS in patients with RCC. **(A)** overall patient population; **(B)** subgroup analysis by various cut-off values of PNI; **(C)** subgroup analysis by various cut-off value determination methods; **(D)** subgroup analysis by various survival analysis types.

The association between PNI and CSS was analyzed based on data from four studies comprising 2,078 patients (17–19, 24). The overall results had HR = 2.95, 95% CI = 1.61–5.39, p<0.001 in REM (**Figure 4** and **Table 5**). Subgroup analysis showed that low PNI was a significant prognostic factor for poor CSS when the cutoff value was ≥45, but the prognostic value was invalid with a cutoff value <45 (**Figure 4** and **Table 5**).

**Table 5:** Results of subgroup meta-analysis for cancer-specific survival.

Variables	No. of studies	No. of patients	Effects model	HR (95%CI)	p	*I* ^2^ (%)	P for heterogeneity
Total	4	2,078	REM	2.95 (1.61-5.39)	<0.001	67.2	0.027
Cut-off value							
<45	1	660	–	1.51 (0.94-2.45)	0.089	–	–
≥45	3	1,418	FEM	4.05 (2.61-6.29)	<0.001	0	0.830
Cut-off value determination							
ROC analysis	2	783	FEM	3.65 (1.95-6.85)	<0.001	0	0.681
Cox model	1	635	–	4.47 (2.42-8.27)	<0.001	–	–
X-tile program	1	660	–	1.51 (0.94-2.45)	0.089	–	–
Survival analysis							
MVA	3	1,443	FEM	2.49 (1.28-4.87)	0.008	59.3	0.086
UVA	1	635	–	4.47 (2.42-8.27)	<0.001	–	–

ROC, receiver operating characteristic; MVA, multivariate; UVA, univariate; FEM, fixed-effects model; REM, random-effects model.

### Sensitivity Analysis

**Figure 5:**
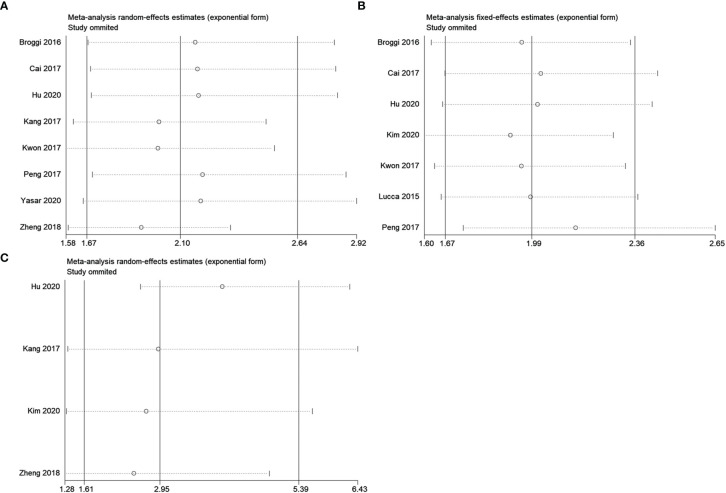
Sensitivity analysis for **(A)** OS, **(B)** PFS/DFS/RFS, and **(C)** CSS in this meta-analysis.

We examined the stability and reliability of pooled HRs and 95% CIs for OS, PFS/DFS/RFS, and CSS based on sensitivity. As shown in **Figure 5**, the conclusions were reliable as the combined data remained substantially unchanged by the removal of any individual study.

### Publication Bias

**Figure 6:**
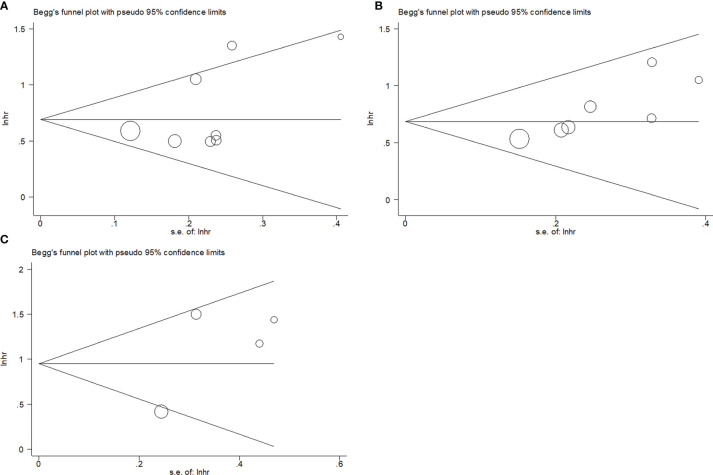
Publication bias test by Begg’s funnel plots for **(A)** OS, **(B)** PFS/DFS/RFS, and **(C)** CSS.

Publication bias was tested using the Begg’s test and funnel plots. The Begg’s p values for OS, PFS/DFS/RFS, and CSS were p = 0.063, p = 0.327, and p = 0.734, respectively. Visual inspection of the funnel plots was symmetrical (**Figure 6**), suggesting that there was no significant publication bias in the meta-analysis.

## Discussion

The pooled analysis of survival data from 10 studies with 4,908 patients showed that reduced PNI associated with poor OS, PFS/DFS/RFS, and CSS in patients with RCC. The results in subgroups stratified by ethnicity, cutoff value, cutoff value determination method, treatment, and survival analysis type were consistent with the overall trend. Sensitivity analysis and publication bias tests confirmed the robustness of the results. Thus, the meta-analysis showed that a low PNI is a significant and reliable prognostic parameter in patients with RCC. PNI could be applied as a promising indicator for survival prediction in RCC.

The PNI is calculated as follows: serum albumin (g/L) + 0.005 × lymphocyte count (per mm^3^) (29). PNI is a parameter that combines the nutritional and inflammatory statuses of patients. The mechanisms of association between low PNI and poor survival outcomes can be explained as follows: First, a low serum albumin level reflects malnutrition in patients with cancer. Malnutrition accounts for 20% of all cancer-related deaths (30). The presence of cancer cachexia is frequently observed, and reduced albumin levels can directly reflect the severity of malnutrition (31). Low pretreatment serum albumin levels are correlated with inferior survival in patients with urothelial carcinoma (32). Moreover, lymphocyte counts reflect antitumor activity in the host. Lymphocytes play a critical role in T cell-related antitumor responses (33). Tumor-infiltrating lymphocytes (TILs) can induce cytotoxic cell death and suppress tumor cell proliferation and migration (34). Based on this evidence, patients with low PNI may suffer from a weakened antitumor response, and therefore, poor survival.

Several recent meta-analyses have also focused on the prognostic ability of PNI in patients with solid tumors (10, 11, 35). Liao et al. reported that lower PNI correlated with unfavorable prognostic factors and poor prognosis in patients with EC, based on a meta-analysis of 3,118 patients (35). Li et al. showed that a low PNI associated with shorter OS in patients with pancreatic cancer (10). Additionally, prognostic significance in their study was not affected by subgroup variables (10). Another meta-analysis demonstrated that a low PNI could predict short- and long-term survival outcomes in patients with nasopharyngeal carcinoma (36). A recent meta-analysis of nine studies indicated that a low PNI status closely correlated with decreased OS in patients with small cell lung cancer. The findings of the present meta-analysis are consistent with those of previous meta-analyses of PNI in other cancer types. As PNI is cost-effective and easily obtained from laboratory tests, PNI can be helpful for clinicians in the management of patients.

In recent years, several studies have found that lymphopenia is a prognostic factor for poor survival in patients with RCC (37, 38). A low PNI represents poor nutritional status and is associated with worse survival in patients with RCC (17, 19, 23, 24). Moreover, the studies included in the present meta-analysis (15–24) indicated inconsistent prognostic value of PNI in RCC, which led to heterogeneity between studies. There are several factors contributing to this. First, the cutoff values of PNI were not uniform in the studies, ranging from 38.5 to 51.62. Therefore, stratification of patients in the low/high PNI groups varied in the included studies. Second, patients received partial or radical nephrectomy and TKIs in different groups, which might have led to selection bias. Patients receiving nephrectomy usually have a good physical condition and non-metastatic disease. However, patients frequently receive TKIs as an adjuvant treatment and have metastatic disease. Malnutrition is less common in patients with non-metastatic RCC than in those with metastatic RCC. Selection bias may exist across studies. Third, all included studies were retrospective in nature. The inherent nature of retrospective studies may lead to heterogeneity, and therefore, the inconsistent results in the included studies. The subgroup analyses by cutoff values of PNI, treatment methods, and metastatic status confirmed the prognostic role of PNI in these subgroups; but the source of heterogeneity should also be acknowledged.

This meta-analysis had some limitations. First, a majority of the studies included were from Asia, lacking data from other regions. Therefore, the prognostic value of PNI in patients with RCC from non-Asian countries should be further confirmed. Second, the methods for determining cutoff and the cutoff values were not uniform in the included studies. Thus, a standard cutoff value for PNI is needed in the clinical settings. Third, the heterogeneity of OS analysis was significant, and selection bias might have been introduced; although sensitivity analysis and publication bias indicated reliability of the results.

## Conclusions

In conclusion, this meta-analysis demonstrated that low PNI associates with shorter survival outcomes in patients with RCC. The prognostic role of PNI is consistent in various patient populations. Furthermore, large-scale studies with standard assessment methods should be conducted to confirm the study findings.

## Data Availability Statement

The original contributions presented in the study are included in the article/**Supplementary Material**. Further inquiries can be directed to the corresponding author.

## Author Contributions

CM and WX designed the study. CM, WX, and JY performed the literature search, literature selection, and data extraction. WM and CW checked the data extraction. ZG and JY statistically analyzed the obtained data. CM, WX, and JY wrote the manuscript. All authors contributed to the article and approved the submitted version.

## Funding

This work was supported by Scientific Research Projects of Jiading District Health Committee (General) (Grant No. 2020-KY-05), Research Projects of Agricultural and Social Undertakings in Jiading District (Grant No. JDKW-2020-0020), and Shanghai Key Specialty Project of Clinical Pharmacy (Grant No. YXZDZK-01). The funders had no role in study design, data collection and analysis, decision to publish, or preparation of the manuscript.

## Conflict of Interest

The authors declare that the research was conducted in the absence of any commercial or financial relationships that could be construed as a potential conflict of interest.

## Publisher’s Note

All claims expressed in this article are solely those of the authors and do not necessarily represent those of their affiliated organizations, or those of the publisher, the editors and the reviewers. Any product that may be evaluated in this article, or claim that may be made by its manufacturer, is not guaranteed or endorsed by the publisher.

## Supplementary Material

Supplementary Figure 1 Forest plot examining the association between PNI and OS in patients with RCC. **(A)** subgroup analysis by various metastatic status of disease; **(B)** subgroup analysis by histological types of RCC.

Supplementary Figure 2 Forest plot examining the association between PNI and PFS/DFS/RFS in patients with RCC. **(A)** subgroup analysis by various metastatic status of disease; **(B)** subgroup analysis by histological types of RCC.

## Abbreviations

PNI, prognostic nutritional index; RCC, renal cell carcinoma; HR, hazard ratio; CI, confidence interval; OS, overall survival; PFS, progression-free survival; DFS, disease-free survival; RFS, recurrence-free survival; CSS, cancer-specific survival; EC, esophageal cancer; PRISMA, Preferred Reporting Items for Systematic Reviews and Meta-Analyses; NOS, Newcastle-Ottawa Scale; REM, random-effects model; FEM, fixed-effects model; ccRCC, clear cell renal cell carcinoma; nccRCC, non- clear cell renal cell carcinoma; TKIs, tyrosine kinase inhibitors; NR, not reported; ROC, receiver operating characteristic; MVA, multivariate; UVA, univariate.
