# Supplementary figures and images for: Prognostic Value of Pretreatment Prognostic Nutritional Index in Patients With Renal Cell Carcinoma: A Meta-Analysis
Source: Front Oncol. 2021 Oct 5;11:719941. doi: 10.3389/fonc.2021.719941 (PMC8523954; doi:10.3389/fonc.2021.719941)

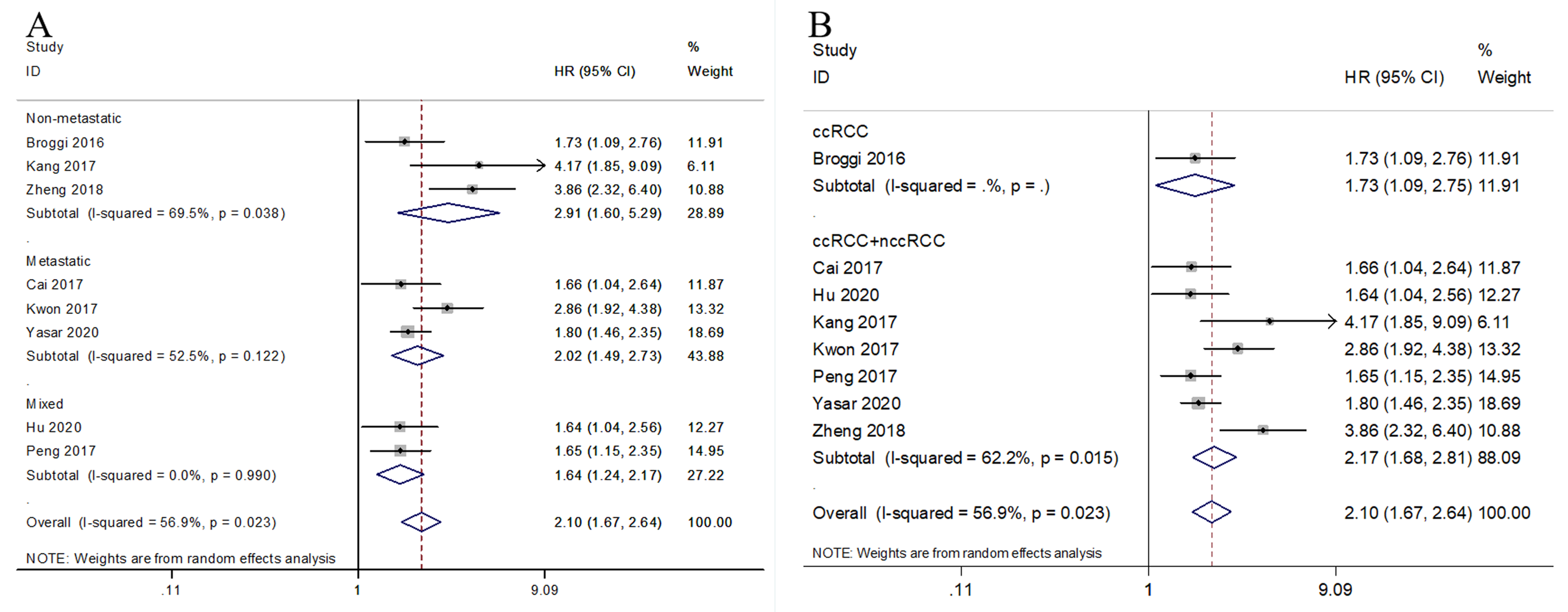

Supplement: Supplementary Figure 1 — Forest plot examining the association between PNI and OS in patients with RCC. (A) subgroup analysis by various metastatic status of disease; (B) subgroup analysis by histological types of RCC. [file Image_1.tif]

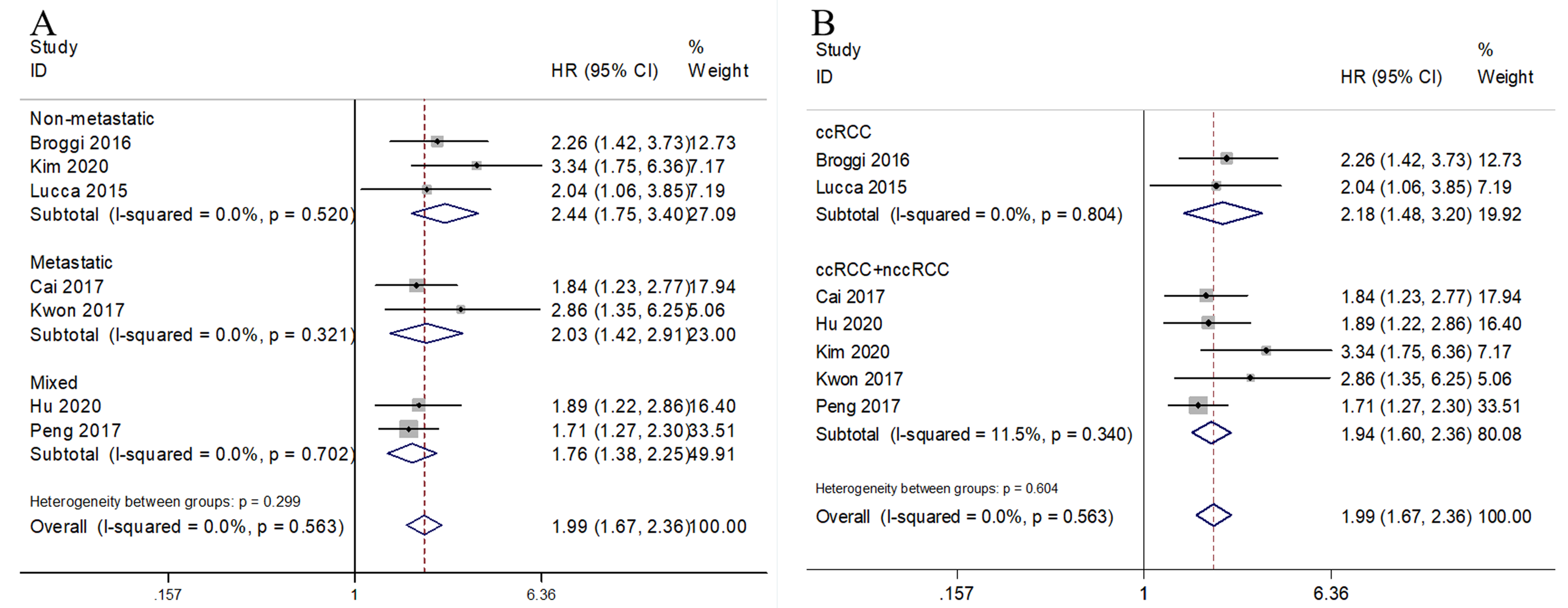

Supplement: Supplementary Figure 2 — Forest plot examining the association between PNI and PFS/DFS/RFS in patients with RCC. (A) subgroup analysis by various metastatic status of disease; (B) subgroup analysis by histological types of RCC. [file Image_2.tif]
